# Comprehensive Analysis of Human Cells Motion under an Irrotational AC Electric Field in an Electro-Microfluidic Chip

**DOI:** 10.1371/journal.pone.0095231

**Published:** 2014-04-15

**Authors:** Clarisse Vaillier, Thibault Honegger, Frédérique Kermarrec, Xavier Gidrol, David Peyrade

**Affiliations:** 1 Univ. Grenoble Alpes, LTM, Grenoble, France; 2 CNRS, LTM, Grenoble, France; 3 Department of Electrical Engineering and Computer Science, Massachusetts Institute of Technology, Cambridge, Massachusetts, United States of Amercia; 4 CEA, Institut de Recherches en Technologies et Sciences pour le Vivant, Grenoble, France; Queen's University at Kingston, Canada

## Abstract

AC electrokinetics is a versatile tool for contact-less manipulation or characterization of cells and has been widely used for separation based on genotype translation to electrical phenotypes. Cells responses to an AC electric field result in a complex combination of electrokinetic phenomena, mainly dielectrophoresis and electrohydrodynamic forces. Human cells behaviors to AC electrokinetics remain unclear over a large frequency spectrum as illustrated by the self-rotation effect observed recently. We here report and analyze human cells behaviors in different conditions of medium conductivity, electric field frequency and magnitude. We also observe the self-rotation of human cells, in the absence of a rotational electric field. Based on an analytical competitive model of electrokinetic forces, we propose an explanation of the cell self-rotation. These experimental results, coupled with our model, lead to the exploitation of the cell behaviors to measure the intrinsic dielectric properties of JURKAT, HEK and PC3 human cell lines.

## Introduction

AC electrokinetic forces have been used in numbers of methods ranging from particle/cell characterization [1], [2], separation [3], [4] or manipulation [5], [6] and can be applied to biosensors, cell therapeutics, drug discovery, medical diagnostics, microfluidic and particle filtration [7] thanks to various designs of electrodes and/or microchannels. These forces induce both liquid and micro-scaled objects motions, namely electro-hydrodynamic (EHD) and dielectrophoretic (DEP) forces. EHD is coupling both linear and non-linear electrokinetic phenomenon that have been discovered and studied in microfluidic channels during the past decade, respectively electrothermal effect (ETE) and AC/induced charged electroosmosis (ACEO/ICEO)[8], [9]. EHD forces create motion of liquid that drags micro-objects along streamlines. Those forces are specific to the electric properties of the suspension media and are difficult to tune in microsystems. On the contrary, DEP has been discovered by Pohl [10] in the 1950's. DEP is a contactless induced force that polarizes micro-objects and induces their motion relatively to the electrodes, providing a non-uniform distribution of the electric field. What is significantly interesting in using DEP to manipulate micro-objects is that its magnitude and direction of the force are directly linked to the frequency and voltage of the applied electric field, which makes the applied force and thus the movement of the object tunable by the electric field properties. There is however a competition between EHD and DEP forces in microsystems [11], [12], which results in a variety of behaviors of objects relatively to the electrodes. Besides understanding the physics of this competition, there has been couple of studies describing the observed motions of micro- and nanoparticles in such microsystems [13], [14]. However, cells are fundamentally different than colloidal particles, either by size, shape, deformability and electrical properties, which results in very different behaviors than the ones previously reported with commercial or engineered particles. For example, cells can present different polarizabilities if alive or dead [15] when applying the same AC fields. Moreover, recent work has reported self-rotation under non rotating fields and the origin of this observation is still unclear [16]. Here, we present a qualitative and quantitative analysis of the induced motion of human cells by non-uniform AC electric fields. Based on the state-of-art comprehensive analysis of colloidal particles motion under such fields, we first report and analyze the motion of three human cells lines when tuning the parameters of the applied electric field. We then suggest possible mechanisms that could lead to those behaviors. We finally exploit those motions to measure the values of the electrical properties of such cells.

### Theory

Castellanos et al. presented a model [12] based on a scaling law approach that described the motion of colloidal particles between planar electrodes. This model described the comprehension of the competition between DEP and EHD forces in the assumption that the electric field distribution is semi-circular and 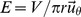 where V is the amplitude of the applied voltage and r is the distance to the center of the gap. Here, we adapt their model to human cells to provide a better understanding of the competition of forces applied on cells and to explain their motions.

### Dielectrophoresis

**Figure 1:**
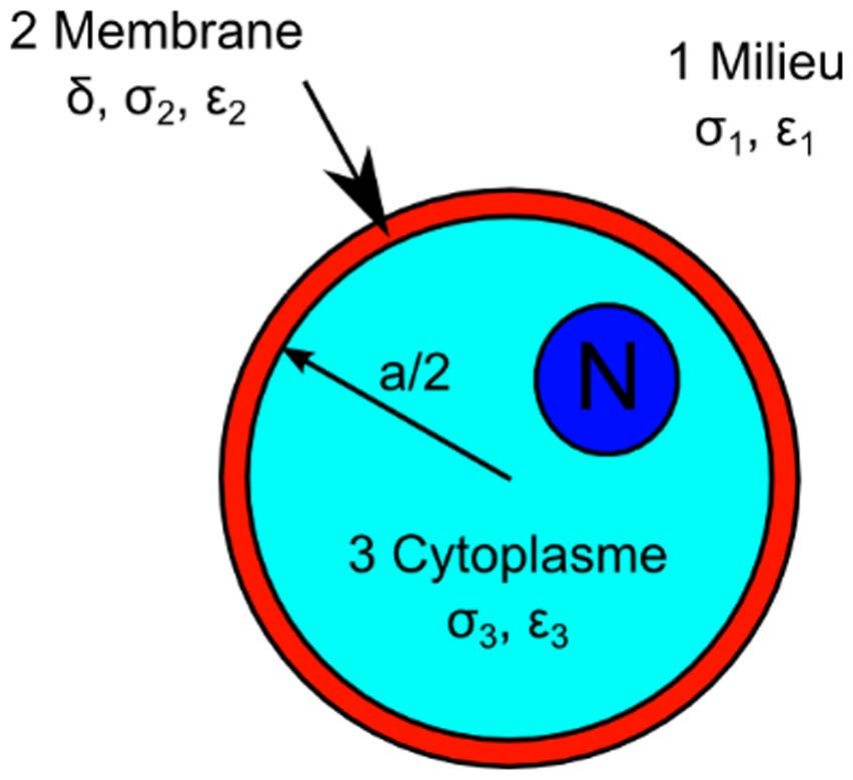
Single shell model of a mamalian cell with dielectric parameters annoted.

Non-uniform electric fields can be used to induce motion of cells. When a cell is suspended in a viable dielectric medium, the applied AC electric field causes the cell to polarize, giving rise to a net dipole moment in the cell. If the electric field is non-uniform, the cell will experience a force. This force is referred to as Dielectrophoresis. By adjusting the experimental conditions, it is possible to move cells towards (positive dielectrophoresis) or away from high field regions (negative dielectrophoresis).The dielectrophoretic force is given in equation (1) [17].
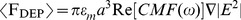
(1)where 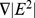 is the gradient of the square of the RMS electric field E, *ω* is the angular velocity of the electric field, a is the cell radius, Re[] indicates the real part and *CMF(ω)* is the Clausius-Mossotti factor (CMF) that translates the relative polarizability of the cell to the medium at a given frequency. The CMF depends on the complex permittivities of the cell and of the medium (permittivity ε_m_, conductivity σ_m_). In the single shell model of a human cell [18], as illustrated in Figure 1, the dielectric properties of a cell are generally expressed with the membrane capacitance *C_mem_* and conductance *G_mem_*. The membrane of mammalian cells is generally poorly conductive and *G_mem_* is usually negligible compared to *C_mem_* [19].

Assuming the membrane thickness δ<<a/2, the general form of the CMF of cells is given in equation (2) [20], [21].
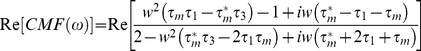
(2)


Where 
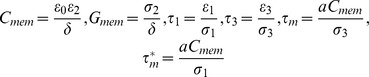
The main assumption of this model is that the cells are spherical in shape. The model must be modified for other geometries, like spheroids or ellipsoids, as reviewed in [22] or in [23].

This model also assumes that the cell is much smaller than the characteristic length of the electrode separation (i.e. to make the dipole approximation). This assumption basically states that the field gradient length scale is much larger than the cell length scale. In most cases when using cells in electro-microsystems, this assumption may not be valid because the electrode gap is in the same order of magnitude than the cell diameter. In this case, the use of a multipole model boosts the magnitude of the polarization factor, but have essentially no influence on the cell crossover frequency and behavior when varying frequencies ore medium conductivity [24]. We therefore proceed with the original core-shell model as a first order approach to describe the cell behaviors. Adapting Castellanos model to the single shell model of human cells, the DEP displacement expresses as shown in equation (3)
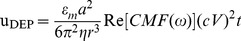
(3)where η is the viscosity of the medium, 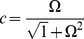 a factor that takes into account the reduction of the voltage in the medium due to electrode polarization (Ω is defined hereafter), V the applied voltage peak to peak, *r* the distance and *t* the duration at which the velocity is evaluated.

**Figure 2:**
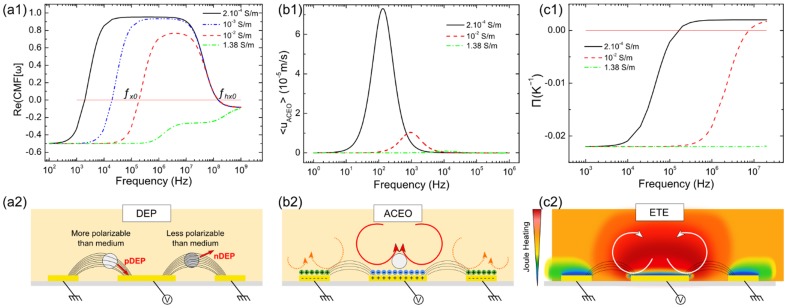
Plots of critical parameters and streamlines induced by AC electrokinetic forces. **(a.1)** Plot of the real part of the Clausius-Mossotti factor for human cells with single shell models (parameters extracted respectively from [27] for HeLa-60) and σ_m_  = 2.10−2 S/m, **(b.1)** Plot of the ACEO mean velocities of the fluid near the electrodes (x = 1 µm) for several conductivities of the fluidic medium for water (ε_f_ = 78) and **(c.1)** Plot of the Π factor as a function of frequency for several conductivities of the medium. Review of predominant forces in presence of AC electric field and the induced motion of liquid and cells: **(a.2)** Dielectrophoresis (DEP) induces attraction (p-DEP) or repelling (n-DEP) of cells from high field region (in co-planar cases, electrode edges), **(b.2)** AC electroosmosis (ACEO) are electrohydrodynamic forces that create convective rolls over the electrodes edges and drag cell with them and **(c.2)** electrothermal effects (ETE).

Figure 2.a1 plots the evolution of the theoretical values of the CMF of 10 µm cells when increasing the medium conductivity. DEP is mostly negative at cell culture medium conductivities (typically PBS or DMEM whose conductivities are around 1 S/m). The classical shape presents a crossover frequency *f*_x0_, where DEP becomes null and changes direction.

Our recent works [25], [26] have used a method to experimentally evaluate the CMF of any polarizable particle, allowing a direct measurement of their dielectrophoretic properties.

### Electrohydrodynamical forces (EHD)

Whereas DEP induces motion of the cell itself, the application of an AC electric field inside a fluid creates two major EHD phenomena, namely ACEO and ETE motions. Those effects will alter the DEP manipulation of cells.

First, AC electroosmosis (ACEO) refers to the flow motion created on the electrodes' surfaces when AC signals are applied. The motion of charges in the electrical double layer induced by the dissymetry of the tangential field will create convection rolls at the electrode interface.

Therefore, there exists an optimal AC frequency at which the product of the electric field and the interface potential, referred as the zeta potential, reaches a maximum. The frequency dependency of fluid motion in co-planar electrodes can be estimated by introducing a non-dimensional frequency Ω given in equation (4) [12], [28].
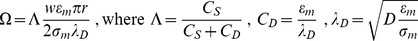
(4)


Where D is the diffusion coefficient of the medium and λ_D_ the Debye layer distance. The value of the Stern layer capacitance C_s_ ∼ 0.007 F.m^-2^ has been used from previous experiments in literature [12]. In the semi-circular electric field approach, the resulting mean velocity induced by ACEO is given in equation (5).
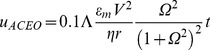
(5)


Figure 2.b1 shows the velocities of 10 µm cells when increasing the medium conductivity, which makes ACEO significantly unobservable at cell culture medium conductivities.

Second, the electrothermal effect (ETE) is observed when a non-uniform electric field is applied over a fluid, Joule heating is produced inside the volume of the fluid, which leads to temperature gradient ∇T in the fluid. This variation produces spatial gradients in the local permittivity and conductivity of the fluid, given as α and β respectively (equation (6)) [11].
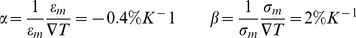
(6)


Furthermore, those gradients generate mobile space charges ρ, in the bulk fluid, following 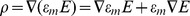 and 
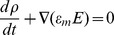
 in AC fields. The time average of the electric force that acts on the fluid through viscosity and leads to fluid transport is given in equation (7).
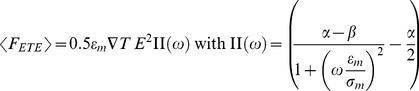
(7)


where the factor Π plays a significant role in the magnitude and direction of the force as shown in Figure 2.c1 for different medium conductivities. When the left term on equation is greater than the right one, Π is positive and the fluid flows from the edge to the center of the electrode. For negative values of Π, the flow pattern is in the opposite direction, as shown in Figure 2.c2. Finally, in the semi-circular approach, the induced displacement of the ETE is given in equation (8).
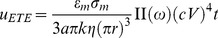
(8)where k is the thermal conductivity of the medium.

Whereas ACEO is a dominant force at small frequencies of the AC field (typically below 10 kHz) and decreases in magnitude when raising the conductivity of the medium, ETE will remain constant at all frequencies and will strongly increase with the conductivity of the medium. On the contrary, the DEP force can change direction in the *f*<10^6^ Hz range frequencies and when the conductivity of the medium is low (σ_m_< 10^-2^ S/m) but will remain negative at higher conductivities.

In the conductivity range of biological medium (σ_m_ ∼ 1 S/m), it has been shown that EHD forces become dominant when rising the voltage [12] but there exists a window of operation in which DEP is still active (V < ∼3 V_pp_) and where biological sample can be manipulated without being injured by heat [29].

The large differences between inorganic particles and cells lead to distinct overall behaviors when immersed in non-uniform AC electric fields. First, size has a cubic square dependency on the DEP force and generally human cells are one or two folds much bigger in diameter than colloidal particles. Second, human cells are mostly composed of water whose permittivity is similar to the one of their suspension media. Moreover, cells exhibit a membrane that separates the interior of the cell, which usually has specific conductivities with all the proteins and the cell apparatus, from the external environment, whose conductivity is buffered. This membrane is permeable to ions and other charged molecules and act as a barrier to the polarization effect of the interior of the cell, until the electric breakdown point where the membrane becomes transparent to the electric field. This complex polarization process gives rise to an opposite behavior than the one observed for uniform dielectric inorganic particles, whose motion under electric fields have been fully supplied by the community.

## Material and Methods

### Cell culture and preparation

PC3, JURKAT and HEK lines were commercially available cell lines and were purchased at the American Type Culture Collection, respectively http://www.lgcstandards-atcc.org/Products/All/CRL-1435.aspx, http://www.lgcstandards-atcc.org/Products/All/TIB-152.aspx, http://www.lgcstandards-atcc.org/Products/All/CRL-1573.aspx. These cells were cultivated in 25 cm^2^ tissue-culture treated flasks (Product 353109, Corning Life Sciences), at 37°C, in 5% carbon dioxide. Both JURKAT and PC3 lines grew in RPMI standard medium, and HEK in DMEM standard medium, all supplemented with 5% of fetal calf serum and penicillin-streptomycin mix (1%). For anchorage-dependent cells, they were collected when confluence reaches 80%, using trypsin-EDTA complex (0.25%, Sigma-Aldrich) during 5 minutes at 37°C. JURKAT cells were collected when clusters reached about fifteen cells, by dynamic pipetting.

Cells were finally suspended in sucrose-dextrose medium (8.5%/0.3%) in deionized water respectively, because of its very low conductivity. DMEM medium was added to adjust the medium conductivity up to the wanted value.

### Cell viability

The cell viability in the sucrose-dextrose medium was average (half of cells die in 5 hours), but the very low conductivity of this medium and its ability to conserve the osmotic pressure viable for cells makes it suitable to perform electrokinetic handling during a limiting amount of time (∼1 hour). The experiment being conducted in less than an hour, the cell viability was not observed to decrease significantly. Based on our experiments, we observed that dielectrophoresis had a damaging effect on cells only under highly stressful conditions, (|E| > 0.150 V/µm) or at low frequencies (ƒ ≤ 1 kHz). Those conditions were avoided during our experiments.

### Microfabrication of chips

The fabrication of the chips was based on glass-electrode and soft lithography technology. Briefly, a glass slide was deposited with a bi-layer Ti:Au 15 nm:135 nm by metal evaporation. The slides were then patterned with AZ1512HS, exposed through a mask with a mask aligner (MJB4, Suss), and etched by Ion Beam Etching (Plassys MU400). Resist was stripped in acetone. For soft lithography, PolyDiMethylSiloxane base was mixed with curing agent in a 10:1 ratio (Sylgard 184, Dow Corning), degassed for 15 minutes to remove air bubbles and cured 5 minutes at 110°C on a prefabricated mold with SU-8.

### Observation of cell motion

The cells were placed on top of 50 µm width interdigitated electrodes spaced by 50 µm in a suspending medium whose conductivity had previously been measured. Motion was recorded through a camera (GiGE, AVT Manta, G-201C) mounted on a modified Leika microscope (INM 20) controlled by a home-made Labview software (National Instrument, Labview 8.0, NI-IMAQ Vision v4.5). The recording of the movie was launched before the electric field was turned on. Measurements were conducted on a minimum of 3 cells and when possible on more cells (up to 15 cells), depending on the effective number of cells ongoing rotation.

### Fits

Fits were performed with Origin8 (OriginLab). The Nonlinear Multiple Variables Fitting tool of Origin was used on the experimental datas and fitting variables were initialized with typical known values (e.g. *C_mem_* are initialized at 5 mF/m). Once fitted, the values obtained for the fitting parameters were used to plot the corresponding curve.

### Rotation speed measurements

The tested frequency and voltage were applied on the electrode. The recorded video was opened with Labview (National Instruments). A new template was defined (generally the entire cell, or part of the cell membrane), directly by taking the "region of interest" on the frame. Depending on the adjustable score (between 1 and 1000, 1000 being a perfect correlation between the template and the original video), the template was tracked on all selected frames. A text file was generated with template center coordinates for each frame, and the angle since the precedent frame. The speed was calculated from the coordinates, as the average rotation from angle datas, during a given number of frames (related to the time by the frame rate). The magnitude of the electric field was calculated as |E| = V_p-p_/e where V_p-p_ was the peak to peak voltage applied on the electrodes and e was the gap between the signal and ground electrodes.

## Results and Discussion

**Figure 3:**
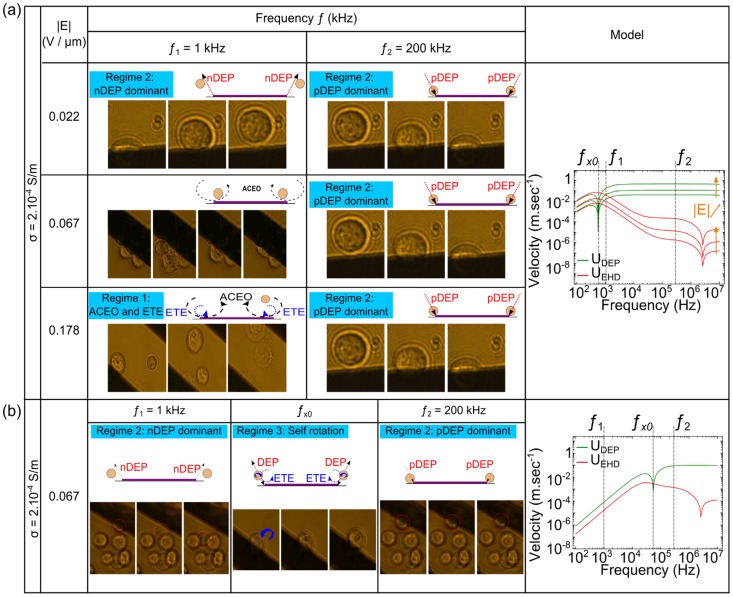
Summary of cells behaviors at (a) σ_m_  =  2.10^−4^ S/m and (b) σ_m_  =  2.10^−2^ S/m. Photographs and schemes illustrate the cell motion for typical frequencies and magnitudes with corresponding graphs of the DEP (U_DEP_, green line) and EHD (U_EHD_, in red) velocities. The velocities were calculated according to the theoretical model presented in the first paragraph and position of the field was taken for x = 1 µm. The boxed text refers to the related paragraph.

Cells were placed on a microfluidic chip on which Au electrodes were activated. As soon as the field was applied, several induced motions were observed depending on the parameters of the applied voltage (frequency, voltage) and on the medium conductivity. Here, for human cells, we report three different regimes, which have been summarized in Fig. 3. We observed cell destruction (regime 1), cell dielectrophoresis (regime 2) or cell self-rotation (regime 3). On this figure we also have plotted the computed velocities of cells induced by DEP and EHD motion based on the model described here before. For the two studied conductivities, DEP determines the overall displacement of cells (regime 2). However, around the inflection point created by the crossover frequency, ETE overpowers DEP and influences cells motion described by regimes 1 and 3. We have also supplied a movie spotting each behavior in Movie S1. The study of cell destruction and dielectrophoresis is conducted on one cell type (HEK epithelial cells), while three different cell types (HEK, Jurkat T-cells and PC3) were studied for the self-rotation phenomenon.

### Regime 1: Cell destruction (ACEO dominant and ETE)

**Figure 4:**
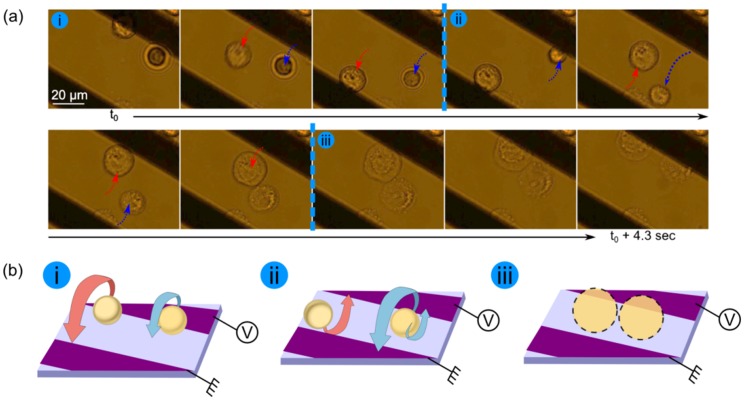
Time-lapse sequence images of cell destruction in conditions of dominant ACEO and ETE (*f*  =  1 kHz, V_pp_  =  8 V, σ_m_  =  2 10^−4^ S/m) (a) picture of HEK cells taken in a microfluidi chip and (b) schema of the observed motions. Cells are dragged in bulk rolls and the membrane rapidly breaks.

At low frequencies (ƒ < 5 kHz) and at σ_m_  =  2.10^−4^ S/m, cells experienced mostly ACEO and were generally attracted to (and maintained above) the edge of the activated electrode. When increasing the magnitude of the electric field (higher voltage or smaller inter-electrodes distance), ETE becomes predominant over other forces and cells are carried away in big convective rolls. In this later case, cell destruction is commonly observed in a few seconds (∼4 s) as shown on Figure 4, either due to the presence of reactive oxygen species or a temperature elevation that has both been observed to lead to cell destruction [30].

### Regime 2: Electrode edge collection or repulsion of cells (DEP dominant)

**Figure 5:**
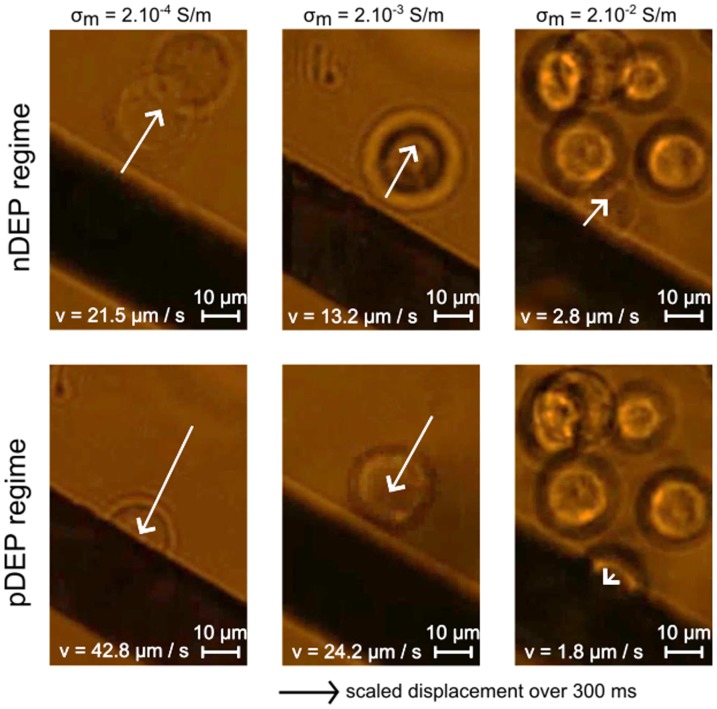
Response of HEK cells to dielectrophoresis for increasing medium conductivities. nDEP and pDEP are applied at *f*  =  1 kHz and *f*  =  200 kHz, respectively. The arrow represents cell motion during 5 frames (300 ms), the picture being the last image. DEP is stronger at low conductivities compared to EHD forces so cells experience larger displacement at higher velocities at low conductivities.

When spanning frequency and voltage, cells are attracted to or repelled from the electrode edges (Figure 5), which is a typical behavior of a dominant DEP regime. Due to the large size of human cells (a ∼> 10 µm) and to the cubic relation of DEP force to the cell radius, cells often experience DEP, even if they are not very close to the electrodes (i.e. *x* > 2a where *x* is the distance from the electrode edges to the cell), which is not necessarily the case for colloidal particles[29].

First, cells are repelled from the electrode edges both in the electrode plane and in the z-axis. Repulsion of cells is foreseen when Re[CMF(ω)] < 0 (negative DEP), which corresponds to a range of low frequencies (*f*<*f_x0_*) and very high frequencies (*f*>*f_hx0_*), as shown on Figure 2.a1. Whereas *f_hx0_* is hardly observable because of difficulties to conduct very high frequencies in microsystems [21], *f_x0_* is commonly observed and depends on cell type, size and medium conductivity [31]. For example, at σ_m_  =  2.10^−4^ S/m, we observe *f_x0_* ∼ 50 kHz for human cells, and at σ_m_  =  10^−2^ S/m, we observe *f_x0_* ∼ 100 kHz. When approaching the crossover frequency, the velocity of cells reduces because the DEP force tends to vanish.

Second, cells are attracted to the electrode edges at higher frequencies (*f_x0_* < *f* < *f_hx0_*), namely the positive DEP regime when Re[CMF(ω)] > 0. Once on the top of the electrode edges, cells do not move anymore and are able to attach on the surface of the glass slide. Moreover, at those frequencies, cells can chain together and create organized assembly as reported previously with human liver cells [32] or 3T3 mouse cells [33]. This pearl chain formation results from the distortion of the electric field distribution by the first attracted cell, creating a high strength distribution at its own edges and therefore attracting another cell to it, and so on.

The influence of the medium conductivity is quantified by measuring cells velocities for both n-DEP and p-DEP regimes. As shown on Figure 5, the magnitude of the DEP force, which is observed *via* the cell motion, is vanishing when increasing the medium conductivity.

### Regime 3: Self-rotation in non-rotating field (ETE)

**Figure 6:**
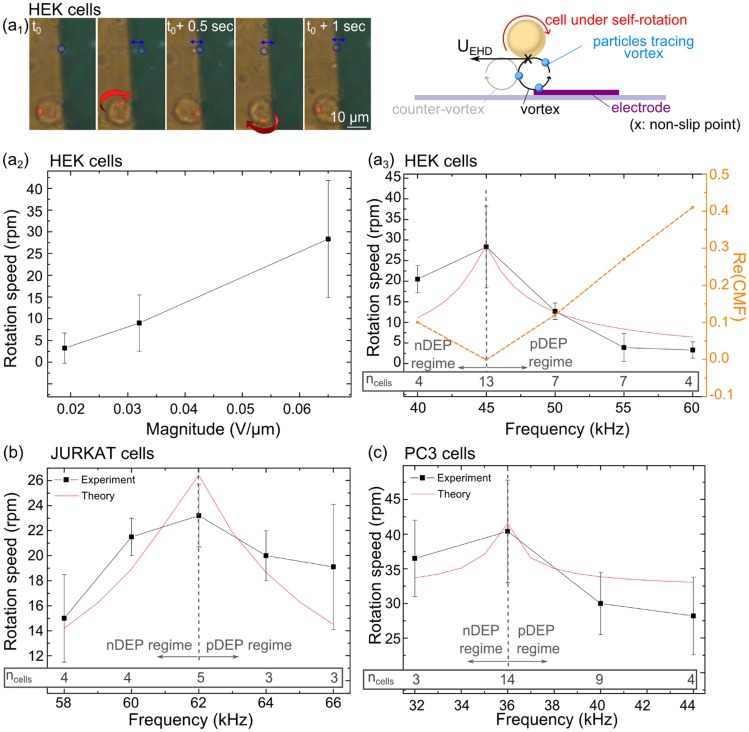
Rotation study of three human cell lines. **(a1)** Time-lapse sequence images of the rotation of HEK cells in the z-axis, in presence of 1 µm polystyrene colloid (highlighted in blue circles). Particles were added to observe medium stream lines. The red circle pinpoints a visible organelle. Rotation is studied at σ_m_  =  2.10^−2^ S/m when varying **(a2)** magnitude of the electric field at *f*  =  45 kHz or **(a3)** frequency at magnitude 0.065 V/µm (V = 10 Vp-p). The dashed line plots the values of |Re[CMF(ω)]| at the same frequencies, bringing out the relation between DEP effect and ETE. Rotation studies of **(b)** of JURKAT cells and **(c)** PC3 cells (electric field magnitude is 0.089 V/µm (V = 4Vp-p) and σ_m_  =  2 10^−2^ S/m.). The inset on the lower part of the graphs shows the number of cells used for each mean value.

Around the crossover frequency (*f*  =  *f_x0_* ± 10 kHz) and at σ_m_ ≤ 10^−2^ S/m, a self-rotation phenomenon of the cells is observed. Cells rotate counterclockwise above the edges of electrodes, with a y-axis of rotation as shown on Figure 6.a_1_. We observed the self-rotation for human cells lines JURKAT, HEK and PC3, as reported in Figure 6.b and 6.c, and the rotation speeds were maximal at the first crossover frequencies *f_xo_*. This phenomenon is particularly surprising since the electric field is non-rotational compared to electro-rotation experiments (ROT). We here report the same behavior than the ones recently observed by Chau *et al*. [16] with melan-a cells or lymphocytes and by Ouyang *et al*. [34] with melanin pigmented cells. In both cases, the origin of such phenomenon was uncertain, as explained by the authors.

Chau *et al*. have hypothesized that the rotational effect of cells is due to the uneven distribution of mass within the cells, thus creating a dipole moment that may drive the cell to rotate continuously. We believe that in that case, the electric field would not penetrate the cell membrane around the crossover frequency *f_x0_*, and prevents a dipole creation inside the cell itself. Ouyang *et al*., have observed the self-rotation of melan-A cells at very high frequencies (f > 10 MHz) and do not report any peak in the rotation speed. They hypothesized the existence of a tangential force in the lower part of the cell that induces a torque and self-rotation. Since their experiments were performed at very high frequencies, there is a low chance to actually have the presence of a crossover frequency (*f_x0_* or f*_hx0_*). However, their experiment shows a quadratic dependency of the rotation speed according to the voltage they used. We believe they have observed a competition of force between positive DEP that attracts the cells towards their active electrodes, and ETE that induces local vortexes at the edges of the electrodes and thus induces self-rotation of cells.

Instead, we suggest that the self-rotation effect is the result of a competition of forces between DEP and the ETE: Around the crossover frequency, cells DEP regimes are being overpowered by ETE forces and at the exact crossover frequency, the DEP force vanishes, letting ETE forces inducing vortexes like induced motion of liquid above the electrode edges, thus dragging the cells into a self-rotation motion.

To support our hypothesis, streamlines of fluid motion were visualized by mixing 1 µm polystyrene colloids to the cells, at the crossover frequency ƒ*_x0_* of cells, as it can be better seen in Movie S1. We observed the rotation of particles just above the edge of the activated electrode, in the z-axis. As overlapped on Figure 6a_1_, the particles were first pushed up away, then attracted to the edge of the electrode (t_0_ + 0.5 sec), and finally ended the cycle by being pushed away again in the other direction (t_0_ + 1 sec). Those observations translate a convective-roll like motion at the edge of electrode. This type of motion was described to be induced by the ETE [13], [29] in co-planar or bi-planar electrodes configuration. Evolution of |Re[CMF(ω)]| values compared to the rotation speed is inverted: the faster the rotation, the less Re[CMF(ω)] as shown on the Figure 6a_3_.

Since ETE forces are liquid induced motions, their influence on the global cell motion is quenched by the magnitude of the DEP force that dominates the cell behavior (attracted or repelled to the electrode edges). However, when approaching the DEP crossover frequency, the DEP force vanishes and the ETE behavior dominates. At this particular frequency, the dependency on voltage raises as shown on Figure 6.a_2_. The cell is much bigger than the convective rolls which induces its self-rotation by slip-free rolling. On the contrary, smaller particles are dragged by the fluid flow and follow the stream in convective rolls motion, as drawn in Figure 6.a_1_.

Following this explanation, the cell rotation *Ω_f_*_x0_ at the crossover frequency *f*_x0_, is expressed with a slip-free rolling condition with the global electrohydrodynamical 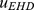 induced velocity on the cell, corrected by a compensation factor 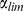 revealing the force competition between ETE and DEP, as shown in equation (9).



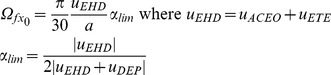
(9)


We have fitted the experimental data observed on the three cell lines and this model. Respective fits are shown on Figure 6. One can observe that the trends are well described by the model, emphasizing the competitiveness competitively between EHD and DEP with a maximum rotation value at the crossover frequency. The fits have been performed when varying σ_3_ and C_mem_ whose values for the three cell lines are reported in Table 1.

**Table 1:** Table of the dielectric parameters for three human cell lines.

Line	Cell Type	σ_3_ (S/m)	ε_3_	*C_mem_* (mF/m^2^)	Cell size (µm)
**HEK**	Adherent cell	0.50 ± 0.1	60	3.27±0.05	15 ± 1.0
**JURKAT**	Circulant cell	0.65 ± 0.12	60	2.38 ± 0.04	15 ± 1.0
**PC3**	Cancerous adherent cell	0.9 ± 0.15	60	3.44 ± 0.02	18 ± 1.0

The cytoplasm conductivity σ_3_ and membrane capacitance *C_mem_* are calculated from experimental fit to the competitive model.

We address the robustness of the rotation method by confronting the measured values of crossover frequencies of the three cell lines determined by the rotation and the more classical observation method, as shown in Movie S1.

We do not observe any changes in cell behavior compared to σ_m_  =  10^−2^ S/m when rising the conductivity of the medium (σ_m_ > 10^−2^ S/m). Indeed, the potential drops within the double layer become null thus the ACEO force fails, DEP is the dominant force over ETE so that DEP is overall the main force acting on the cells unless around the crossover frequency as described in the last paragraphs.

## Conclusion

AC electrokinetic forces have been reported to identify and sort cells according to their dielectric differences and we have presented here the behaviors of three human cells lines when placed in non-uniform electric field. We have analyzed and reported the influence of key parameters of the field that radically change cell motions and shown how to take advantage of those behaviors to characterize and/or handle human cells by AC fields in microfluidic channels. Our analysis gives a sense to the observations reported by several other works related to human cells. We believe that our results will help the community to better understand their experimental observations when handling human cells, to design enhanced experiments when using AC fields to characterize or handle cells and finally to allow the establishment of an accurate database of human cell dielectric properties for accurate cell detection and sorting.

## Supporting Information

Movie S1 **The movie shows the different behaviors of JURKAT cells reported in the article.** Videos are displayed in real time.
(AVI)
